# Supplementary material for: Mechanism of spirometry associated gastro-esophageal reflux in individuals undergoing esophageal assessment
Source: PLoS One. 2023 Sep 13;18(9):e0291445. doi: 10.1371/journal.pone.0291445 (PMC10499194; doi:10.1371/journal.pone.0291445)

PLOS ONE

Mechanism of spirometry associated gastro-esophageal reflux in subjects undergoing esophageal assessment

--Manuscript Draft--

**Manuscript Number:**	PONE-D-23-07360
**Article Type:**	Research Article
**Full Title:**	Mechanism of spirometry associated gastro-esophageal reflux in subjects undergoing esophageal assessment
**Short Title:**	Mechanism of spirometry associated gastro-esophageal reflux
**Corresponding Author:**	Jerry Zhou, Ph.D.<br>Western Sydney University<br>Campbelltown, NSW AUSTRALIA
**Keywords:**	spirometry, lung function, acid reflux, gastro-esophageal reflux, FEV1, lower esophageal sphincter, impedance manometry
**Abstract:**	Variability during spirometry can persist despite control of technical and personal factors. We have previously shown spirometry can provoke gastro-esophageal reflux in a susceptible population. In this study, we aim to determine the mechanisms of spirometry induced reflux. Twenty four (24) participants with reflux symptoms presenting for esophageal assessment were recruited. Participants performed two sets of spirometry separated by a 10-minute rest period, during which fluid flow dynamics and esophageal function were evaluated using high-resolution impedance esophageal manometry. The assessment compared baseline manometric parameters their equivalent values during the first set of spirometry, during the resting period, and during the second set of spirometry. Impedance values, a measure of fluid quantity, during each spirometry set and the recovery period were significantly higher compared with the baseline within the pan-esophagus and hypopharynx region. Furthermore, the lower esophageal sphincter resting pressure was significantly reduced after the first spirometry set (13.6 ±8.8 mmHg) when compared to baseline (22.5 ±13.3 mmHg). A reduction in spirometry parameters; FEV1 (0.14 ±0.24L, P=0.042) and PEFR (0.67 L/s, P=0.34), was also noted in spirometry set 2 compared to set 1. This study demonstrates that spirometry manoeuvres can cause gastro-esophageal reflux through intra-esophageal pressure differentials and a temporary weakening of the lower esophageal sphincter.
**Order of Authors:**	Matthew Xu
	John Brannan, PhD
	Vincent Ho, MBBS, PhD
	Jerry Zhou, Ph.D.
**Additional Information:**	
**Question**	**Response**
**Financial Disclosure**<br><br>Enter a financial disclosure statement that describes the sources of funding for the work included in this submission. Review the submission guidelines for detailed requirements. View published research articles from *PLOS ONE* for specific examples.<br><br>This statement is required for submission and **will appear in the published article** if	The author(s) received no specific funding for this work.

the submission is accepted. Please make sure it is accurate.<br><br>**Unfunded studies**<br>Enter: *The author(s) received no specific funding for this work.*<br><br>**Funded studies**<br>Enter a statement with the following details:<br>• Initials of the authors who received each award<br>• Grant numbers awarded to each author<br>• The full name of each funder<br>• URL of each funder website<br>• Did the sponsors or funders play any role in the study design, data collection and analysis, decision to publish, or preparation of the manuscript?<br>• **NO** - Include this sentence at the end of your statement: *The funders had no role in study design, data collection and analysis, decision to publish, or preparation of the manuscript.*<br>• **YES** - Specify the role(s) played.<br><br>* typeset	
**Competing Interests**<br><br>Use the instructions below to enter a competing interest statement for this submission. On behalf of all authors, disclose any competing interests that could be perceived to bias this work—acknowledging all financial support and any other relevant financial or non-financial competing interests.<br><br>This statement is required for submission and **will appear in the published article** if the submission is accepted. Please make sure it is accurate and that any funding sources listed in your Funding Information later in the submission form are also declared in your Financial Disclosure statement.<br><br>View published research articles from *PLOS ONE* for specific examples.	The authors have declared that no competing interests exist.

**NO authors have competing interests**<br><br>Enter: *The authors have declared that no competing interests exist.*<br><br>**Authors with competing interests**<br><br>Enter competing interest details beginning with this statement:<br><br>*I have read the journal's policy and the authors of this manuscript have the following competing interests: [insert competing interests here]*<br><br>* typeset	
**Ethics Statement**<br><br>Enter an ethics statement for this submission. This statement is required if the study involved:<br><br>• Human participants<br>• Human specimens or tissue<br>• Vertebrate animals or cephalopods<br>• Vertebrate embryos or tissues<br>• Field research<br><br>Write "N/A" if the submission does not require an ethics statement.<br><br>General guidance is provided below. Consult the submission guidelines for detailed instructions. **Make sure that all information entered here is included in the Methods section of the manuscript.**	The study was approved by the South West Sydney Local Health District Ethics Committee (2020/ETH03098). All participants provided written consent to take part in this study and the use of their medical record for research.

Powered by Editorial Manager® and ProduXion Manager® from Aries Systems Corporation

**Format for specific study types**<br><br>**Human Subject Research (involving human participants and/or tissue)**<br>• Give the name of the institutional review board or ethics committee that approved the study<br>• Include the approval number and/or a statement indicating approval of this research<br>• Indicate the form of consent obtained (written/oral) or the reason that consent was not obtained (e.g. the data were analyzed anonymously)<br><br>**Animal Research (involving vertebrate animals, embryos or tissues)**<br>• Provide the name of the Institutional Animal Care and Use Committee (IACUC) or other relevant ethics board that reviewed the study protocol, and indicate whether they approved this research or granted a formal waiver of ethical approval<br>• Include an approval number if one was obtained<br>• If the study involved *non-human primates*, add *additional details* about animal welfare and steps taken to ameliorate suffering<br>• If anesthesia, euthanasia, or any kind of animal sacrifice is part of the study, include briefly which substances and/or methods were applied<br><br>**Field Research**<br><br>Include the following details if this study involves the collection of plant, animal, or other materials from a natural setting:<br>• Field permit number<br>• Name of the institution or relevant body that granted permission	
**Data Availability**<br><br>Authors are required to make all data underlying the findings described fully available, without restriction, and from the time of publication. PLOS allows rare exceptions to address legal and ethical concerns. See the PLOS Data Policy and FAQ for detailed information.	Yes - all data are fully available without restriction

A Data Availability Statement describing where the data can be found is required at submission. Your answers to this question constitute the Data Availability Statement and **will be published in the article**, if accepted.<br><br>**Important:** Stating 'data available on request from the author' is not sufficient. If your data are only available upon request, select 'No' for the first question and explain your exceptional situation in the text box.<br><br>Do the authors confirm that all data underlying the findings described in their manuscript are fully available without restriction?	
**Describe where the data may be found in full sentences. If you are copying our sample text, replace any instances of XXX with the appropriate details.**<br><br>• If the data are **held or will be held in a public repository**, include URLs, accession numbers or DOIs. If this information will only be available after acceptance, indicate this by ticking the box below. For example: *All XXX files are available from the XXX database (accession number(s) XXX, XXX.*).<br>• If the data are all contained **within the manuscript and/or Supporting Information files**, enter the following: *All relevant data are within the manuscript and its Supporting Information files.*<br>• If neither of these applies but you are able to provide **details of access elsewhere**, with or without limitations, please do so. For example:<br><br>*Data cannot be shared publicly because of [XXX]. Data are available from the XXX Institutional Data Access / Ethics Committee (contact via XXX) for researchers who meet the criteria for access to confidential data.*<br><br>*The data underlying the results presented in the study are available from (include the name of the third party*	The data that support the findings of this study are available from the corresponding author upon reasonable request.

and contact information or URL).<br>• This text is appropriate if the data are owned by a third party and authors do not have permission to share the data.<br><br>* typeset	
Additional data availability information:	Tick here if the URLs/accession numbers/DOIs will be available only after acceptance of the manuscript for publication so that we can ensure their inclusion before publication.

Manuscript

Click here to access/download;Manuscript;Manuscript_Zhou2023.docx

Mechanism of spirometry associated gastro-esophageal reflux in subjects undergoing esophageal assessment

Matthew Xu[1], John D. Brannan[2], Vincent Ho[1,3], Jerry Zhou[1]

1 School of Medicine, Western Sydney University, Campbelltown, NSW, Australia,
2 Department of Respiratory & Sleep Medicine, John Hunter Hospital, New Lambton, NSW, Australia
3 Department of Gastroenterology, Campbelltown Hospital, Campbelltown, NSW, Australia

Corresponding author
Jerry Zhou
Building 30, School of Medicine Western Sydney University
Goldsmith Av, Campbelltown 2560
Sydney, NSW, Australia
j.zhou@westernsydney.edu.au

Word Count
2,402 words

Abstract

Variability during spirometry can persist despite control of technical and personal factors. We have previously shown spirometry can provoke gastro-esophageal reflux in a susceptible population. In this study, we aim to determine the mechanisms of spirometry induced reflux. Twenty four (24) participants with reflux symptoms presenting for esophageal assessment were recruited. Participants performed two sets of spirometry separated by a 10-minute rest period, during which fluid flow dynamics and esophageal function were evaluated using high-resolution impedance esophageal manometry. The assessment compared baseline manometric parameters their equivalent values during the first set of spirometry, during the resting period, and during the second set of spirometry. Impedance values, a measure of fluid quantity, during each spirometry set and the recovery period were significantly higher compared with the baseline within the pan-esophagus and hypopharynx region. Furthermore, the lower esophageal sphincter resting pressure was significantly reduced after the first spirometry set (13.6 ±8.8 mmHg) when compared to baseline (22.5 ±13.3 mmHg). A reduction in spirometry parameters; FEV_1 (0.14 ±0.24L, P=0.042) and PEFR (0.67 L/s, P=0.34), was also noted in spirometry set 2 compared to set 1. This study demonstrates that spirometry manoeuvres can cause gastro-esophageal reflux through intra-esophageal pressure differentials and a temporary weakening of the lower esophageal sphincter.

Introduction

Spirometry is the most common pulmonary function test for the diagnosis and monitoring of respiratory disorders. Key component of spirometry testing is the forced vital capacity (FVC) maneuver, which can be split into two distinct manouvres; first is inspiration to total lung capacity (TLC) followed by forced expiration to residual volume. Spirometry is considered both safe and reproducible(1). However, variability during spirometry can persist in some patients despite control for technical factors and patient technique has been optimized. We have recently determined that small but significant variation in spirometry can occur in patients with active gastric esophageal reflux (GER)(2). We observed an FVC maneuver can provoke gastro-esophageal reflux (GER) in 45% of individuals with GER symptoms. Within this subgroup, GER events were observed during FVC maneuver and the recovery period over 10 minutes. Furthermore, repeat spirometry testing after the recovery period saw a significant decrease in spirometry parameters; peak expiratory flow rate (PEFR) and forced expiratory volume (FEV_1).

However, it is unclear what the direct or indirect role of reflux is on spirometry and its variability. Cough-like expiratory efforts termed “deflation cough” was first documented during FVC maneuver in individuals with GER symptoms(3). Following on from this, inhibition of deflation cough was demonstrated through pre-treatment with anti-reflux medication(4). These studies suggest a causative role of acidic GER in spirometry variability.

We aimed to further determine the mechanism of spirometry induced GER using high-resolution impedance manometry (HRIM). Our primary objectives were to evaluate fluid flow during FVC using high-resolution impedance manometry (HRIM) and determine possible mechanisms that could predispose the individual to GER during the recovery period and subsequent spirometry attempts.

Materials and Methods

Patient Selection

The study was approved by the South West Sydney Local Health District Ethics Committee (2020/ETH03098). All participants provided written consent to take part in this study and the use of their medical record for research. From September 2021 – December 2022, patients aged 18 to 90 attending the Gastrointestinal Motility Clinic at Camden Hospital, NSW Australia for a HRIM and ambulatory 24hr pH study were recruited. The participants in this study are representative of a population that experiences frequent GER symptoms and/or are diagnosed with gastro-esophageal reflux disease (GERD). Patients with GERD were defined by having an esophageal pH <4 for ≥5.5% of total study time and a DeMeester score ≥14.72 during the 24-hour ambulatory pH study. All patients were off proton pump inhibitors for at least 3 days prior to study.

Exclusion criteria for study participants were; a history of prior gastric or esophageal surgery, history of nasal and eye surgery, a major esophageal motor disorder by Chicago Classification(5) (i.e. achalasia, aperistalsis, jackhammer, distal esophageal spasm), and pregnant and lactating females. Patients who currently used opioid analgesics and anticholinergic drugs were excluded, but patients who were taking a low dose of tricyclic antidepressants, for example nortriptyline (up to 50 mg/d) or amitriptyline (up to 25 mg/d) were eligible to participate provided these medications were commenced 3 months before the study. Patients with technically limited HRIM or ambulatory pH studies were excluded.

Esophageal Function Testing

A solid-state HRIM catheter consisting of 36 circumferential sensors spaced at 1 cm intervals (Medtronic Inc., Shoreview, MN) and 18 impedance sensors spaced at 2 cm intervals was transnasally intubated with the patient in a seated position. After a landmark calibration phase (20 – 60 seconds), ten 5 mL 0.9% saline swallows spaced 20 to 30 seconds apart were completed. Following standard esophageal assessment, another landmark calibration was performed before participants were asked to perform two sets of spirometry maneuvers separated by a 10 minute recovery period while intubated with the HRIM catheter. Thermal compensation was applied at the end of the studies using

ManoView Software. Following HRIM studies, patients with suspected GERD underwent ambulatory pH studies as per standard clinical protocol. A multichannel intraluminal impedance pH catheter (Medtronic Inc., Shoreview, MN) was transnasally intubated with positioning confirmed based on the manometrically identified lower esophageal sphincter (LES). The catheter consisted of a pH sensor positioned 5 cm above the LES. Participants were asked to swallow 10 mL 0.9% saline solution prior before measuring baseline impedance values and initiating the spirometry study. The salt content of salient solution reduces the impedance level (kΩ), visually represented by purple overlay. All esophageal function studies were reviewed using AccuView 5.2 and ManoView ESO 3.0.1 (Medtronic).

Spirometry

Spirometry was conducted using Spiroscout (Ganshorn Medizin Electronic, Niederlauer, Germany) and laptop computer in accordance with the American Thoracic Society/European Respiratory Society (ATS/ERS) spirometry protocol(6) with the addition of the intubated impedance high-resolution manometry catheter. Attempt was made to meet ATS/ERS criteria for each spirometry sets however, this was not achievable in all studies due to the participant's response to the manometry catheter and its impact on performing spirometry. Participants were instructed to inhale to total lung capacity before performing a FEV maneuver and expiring to residual volume, for a minimum of three attempts and no more than five. We recorded the forced vital capacity (FVC), forced expiratory volume in 1 second (FEV1) and peak expiratory flow rate (PEFR). Subjects then had a 10 minute recovery period, before performing a second set of spirometry. Spirometry studies were reviewed using Ganshorn LFX 1.8 (Ganshorn).

Data Collection

Non-identifiable information were collected for this study. Impedance values were obtained with the "Smart Mouse" selection tool in ManoView software. The pan-esophageal impedance was defined by the segment between the manometrically defined upper esophageal sphincter (UES) and LES. The

average pan-esophageal impedance value was calculated using an area 1 cm below the UES and 1 cm above the LES over a specified period of time. We defined the hypopharynx as the area 1 cm above the manometrically defined UES. The average impedance value at hypopharynx was calculated using area 1 cm above the UES over a specific period of time. Baseline impedance and LES pressure values were measured from the average over a 20 second period chosen based on the absence of detected swallows. The pre-spirometry baseline values were measured during a second landmark calibration period immediately before initiation of spirometry study. The post-spirometry recovery values were measured following the 10-minute recovery period after 1st spirometry set. Impedance values were also measured during each FVC maneuver and averaged for each spirometry set.

Statistical Analysis

Continuous data were reported as mean with SD or median with range. Categorical data were summarized as frequencies and percentages. The differences between continuous variables were assessed using analysis of variance for multiple group comparisons or the Kruskal-Wallis test to compare medians among the groups when nonparametric tests were indicate. Student *t* test or Wilcoxon tests were used for comparisons between 2 groups. Expiratory Disproportion Index (EDI) was used as an indicator for laryngotracheal stenosis (stenosis EDI > 50); possible response to the presence of spirometry-induced reflux. EDI was calculated using this formula: EDI = FEV_1/PEFR × 100)(7). Statistical analyses were completed using SPSS 25.0 for Windows (SPSS Inc., Chicago, IL, USA).

Results

Patient Characteristics, Manometric, and pH study Findings

The patient population (n=24) was predominantly female with a mean of 54 years of age (22 – 73 yrs) with the majority not having a hiatus hernia or a respiratory disorder (**Table 1**). Approximately, half had normal esophageal function during the high resolution impedance manometry.

Impedance Data

Baseline impedance levels in the esophagus and hypopharynx was observed at 2.52 ±0.74 kΩ and 3.59 ±2.20 kΩ, respectively (**Table 2**). Pan-esophageal impedance was lower than hypopharynx impedance in all test conditions. Impedance levels were significantly reduced in both locations during spirometry sets and recovery period, suggestive of increased liquid refluxate throughout the study.

Impedance levels were lowest during Spirometry Set 2. In the pan-esophagus, Set 2 impedance was significantly lower than impedance measured at baseline, recovery period and Set 1. The recovery period shows a trend towards restoring impedance to baseline levels but this was not statistically in the esophagus or above the UES.

Manometry Data

Pan-esophageal pressurization dynamics were noted during FVC maneuvers and resting period (**Fig. 1**). At FVC, inspiration to TLC is characterized by diaphragmatic contraction and esophageal extension, leading to negative trans-esophageal pressurization (peak value -21.4 ±7.58 mmHg) relative to gastric pressure. Expiration to FVC generated large positive gastric pressures that prorogated back into the esophagus (peak value 122.3 ±31.3 mmHg). The LES and UES were hypertensive for the duration of the expiration maneuver (mean 217 ±71.7 mmHg and 258.3 ±78.7 mmHg, respectively).

Comparison of LES pressures showed significantly (*p*<0.0001) lower LES resting pressure during the recovery period after the first set of spirometry (13.6 ±8.8 mmHg) compared with baseline LES resting pressure (22.5 ±13.3 mmHg) (**Fig. 2**). Normal LES resting pressure range is between 12 to 43 mmHg(8). There was no significant difference in UES resting pressures pre-spirometry (57.9 ±18.4 mmHg) and post-spirometry (56.2 ±20.2 mmHg).

Spirometry Data

Eight (8) patients were unable to produce three reproducible FVC attempts per set, due to discomfort from the catheter or experienced a coughing episode. The remaining 16 of 24 (66.7%) participants successfully produce two sets of spirometry with each containing at least 3 reproducible spirometry attempts (**Table 3**).

Comparison of these spirometry sets showed significant reductions in the mean FEV_1 and PEFR values in spirometry Set 2 compared with those spirometry Set 1. Similarly, the EDI values were significantly higher in the second spirometry set compared to Set 1 and suggested laryngotracheal stenosis.

Discussion

The present findings show the mechanisms behind spirometry induced GER, which was first observed in our previous study in patients with reflux symptoms(2). Our data shows that refluxate can be induced by FVC as a result of the pan-esophageal pressure differentials generated during the maneuver. Furthermore, repeat FVC attempts can temporarily weaken LES tone and predispose the esophagus and hypopharynx to reflux over the course of the recovery period and subsequent spirometry set. The induced refluxate has a detrimental effect on spirometry reproducibility, with lower average FEV_1 and PEFR were noted in Set 2 compared with Set 1.

Tiller and Simpson were the first to measure the peak esophageal pressure during FVC at 102 ± 34 cmH_2O (or 75 ± 25 mmHg)(9). They speculated that gastric content could be propelled upwards into the esophagus by pressures generated during intra-abdominal contractions. We observed similar high intra-esophageal pressures during FVC and was able to confirm, using impedance fluid flow, the retrograde movement of refluxate into the esophagus and hypopharynx as a result of repeat FVC

maneuvers. Interestingly, spirometry induced GER events were more prominently observed in the current study than our previous study [2]. This is most likely attributed to impedance sensors detecting all reflux events (acidic, weakly acidic, and non-acidic reflux), while ambulatory pH probe used in our previous study only detected acidic reflux.

In healthy adults, during activities that increase intra-abdominal pressure (e.g. deep inspiration, forced expiration, trunk flexion) the right crus of the diaphragm contract to increase pressure on the LES, thereby preventing GER. However, physiologic stressors such as exercise or respiratory disease (e.g. cough) can exert intra-thoracic and esophagus pressure fluctuations that exceeds the resting LES pressure, overcoming the gastroesophageal barrier to result in reflux(10, 11). Our findings provide novel evidence to support this theory, showing a temporary reduction in LES resting tone following FVC. This predisposes the patient to GER during the recovery period and second set of FVC. The cause of the LES weakening is unknown, but we suspect repeat changes in the intra-esophagus pressure gradient coupled with a susceptible population could lead to LES fatigue. Alternatively, FVC may increase the frequency of transient LES relaxation (TLESR), which is an abrupt decrease in the pressure of the LES that is not the result of deglutition or secondary peristalsis. TLESRs are primarily triggered by unconstrained air, e.g. belch, which is in accordance with a physiologic role for TLESRs in venting the stomach. Gastric pressure changes as a result of FVC may also trigger TLESR responses.

Our comparison of parameters in spirometry Set 1 with Set 2 found a reduction in FEV_1 and PEFR in the second set. Although a lower FEV_1 and PEFR is commonly due to poorer patient effort, a possible explanation is reduction of large airway caliber, for instance laryngotracheal stenosis (LTS)(12). A previous study(7) was able to reliably differentiate between stenosis and non-stenosis cases using the EDI threshold of >50 with a sensitivity of 95.9% and specificity of 94.2%. Our study cohort started with an EDI below threshold in Set 1 of 47.7 but increased to an EDI of 54.1 by spirometry Set 2. Maronian *et al.*(13) showed that laryngopharyngeal reflux is a contributing factor if not causative factor in

subglottic stenosis (SGS) after finding that all SGS patients tested were positive for reflux. Other studies are less conclusive. In patients with idiopathic progressive subglottic stenosis there was little reflux symptoms or response to anti-reflux medication(14). Our data suggests weakly acidic and non-acidic reflux can also induce EDI above LTS threshold and may be the cause of discrepancies in the role of laryngopharyngeal reflux in stenosis.

There are limitations to our study. Standard HRIM procedure can cause discomfort and stress in some patients. Although there is no documented data on the impact of intubated nasogastric catheters on the individual’s spirometry performance, it is likely to negatively affect results hence the spirometry results may not reflect the actual lung capabilities of our participants. Furthermore, this patient cohort was selected to maximize the likelihood reflux events during spirometry. Hence, these patients have not undergone formal lung function assessment, limiting interpretation of our study findings in lung function patients. Whether gastric refluxate entered the laryngotracheal region was inferred from EDI changes but not directly observed on manometry. Future studies will aim to demonstrate reflux in a population with respiratory symptoms, and use a non-invasive imaging technique, such as gastric labelling fluoroscopy, to detect fluid movement into the laryngotracheal region.

The implications of our study are possibly far-reaching given the prevalence of spirometry in routine lung function clinics. Consequently, this data suggests respiratory physicians should be aware of a patient history with reflux and current reflux activity when interpreting spirometry results to account for the effects of GER. Conversely, aberrant FEV_1 and PEF signals during pulmonary function testing may be informative of latent reflux activity. In conclusion, FVC maneuvers during spirometry causes GER through intra-esophageal pressure differentials and immediately afterward by a temporary weakening of the LES. Understanding the mechanisms of spirometry induced reflux may explain variability in spirometry that may be independent of patient effort of patient, especially those predisposed to GER.

Acknowledgements

References

1. Miller MR, Crapo R, Hankinson J, Brusasco V, Burgos F, Casaburi R, et al. General considerations for lung function testing. Eur Respir J. 2005;26(1):153-61.
2. Zhou J, Teo M, Ho V, Brannan JD. Prevalence and effects of gastro-oesophageal reflux during spirometry in subjects undergoing reflux assessment. PLoS One. 2020;15(2):e0229250.
3. Lavorini F, Fontana GA, Chellini E, Magni C, Pistolesi M, Widdicombe J. Respiratory expulsive efforts evoked by maximal lung emptying. Chest. 2011;140(3):690-6.
4. Chellini E, Lavorini F, Campi G, Mannini C, Fontana GA. Effect of an anti-reflux medical device in the control of deflation cough: A placebo-controlled comparative study with an antacid drug in chronic coughers. Pulm Pharmacol Ther. 2015;33:11-4.
5. Kahrilas PJ, Bredenoord AJ, Fox M, Gyawali CP, Roman S, Smout AJ, et al. The Chicago Classification of esophageal motility disorders, v3.0. Neurogastroenterol Motil. 2015;27(2):160-74.
6. Miller MR, Hankinson J, Brusasco V, Burgos F, Casaburi R, Coates A, et al. Standardisation of spirometry. Eur Respir J. 2005;26(2):319-38.
7. Nouraei SA, Nouraei SM, Patel A, Murphy K, Giussani DA, Koury EF, et al. Diagnosis of laryngotracheal stenosis from routine pulmonary physiology using the expiratory disproportion index. Laryngoscope. 2013;123(12):3099-104.
8. Yadlapati R, Kahrilas PJ, Fox MR, Bredenoord AJ, Prakash Gyawali C, Roman S, et al. Esophageal motility disorders on high-resolution manometry: Chicago classification version 4.0©. 2021;33(1):e14058.
9. Tiller NB, Simpson AJ. Effect of spirometry on intra-thoracic pressures. BMC Res Notes. 2018;11(1):110.
10. Clark CS, Kraus BB, Sinclair J, Castell DO. Gastroesophageal reflux induced by exercise in healthy volunteers. JAMA. 1989;261(24):3599-601.
11. Ayazi S, DeMeester SR, Hsieh CC, Zehetner J, Sharma G, Grant KS, et al. Thoraco-abdominal pressure gradients during the phases of respiration contribute to gastroesophageal reflux disease. Dig Dis Sci. 2011;56(6):1718-22.
12. Lorenz RR. Adult laryngotracheal stenosis: etiology and surgical management. Curr Opin Otolaryngol Head Neck Surg. 2003;11(6):467-72.
13. Maronian NC, Azadeh H, Waugh P, Hillel A. Association of laryngopharyngeal reflux disease and subglottic stenosis. Ann Otol Rhinol Laryngol. 2001;110(7 Pt 1):606-12.
14. Valdez TA, Shapshay SM. Idiopathic subglottic stenosis revisited. Ann Otol Rhinol Laryngol. 2002;111(8):690-5.

Figure 1. High-resolution impedance manometry topography plot (A, C, E, G); the intra-esophageal pressure is assessed in relation to time and distance. An impedance overlay (B, D, F, H); purple hue denotes fluid flow by impedance. Study time-points 1) Pre-spirometry esophageal pressures 2) A forced vital capacity maneuver during Spirometry Set 1. 3) Post-spirometry baseline during recovery period after Spirometry Set 1. 4) A forced vital capacity maneuver during Spirometry Set 2. UES, upper esophageal sphincter; LES, lower esophageal sphincter.

Figure 2. Resting lower esophageal sphincter pressures (mmHg) before and after spirometry (n=24). Lower esophageal sphincter resting pressure was significantly lowered after spirometry ($p<0.0001$).

Table 1. Patient characteristics and results from esophageal testing.

**Clinical data**	**N=24**
Female	18 (75%)
Age [mean (range)] (y)	54 (22 – 73)
No history of respiratory disorders	20 (83%)
History of asthma (treated/non-symptomatic)	4 (17%)
Current smoker	0 (0%)
**High-resolution impedance manometry results**	
No hiatal hernia	15 (62%)
Hiatal hernia [median (IQR)] (cm)	2.4 (1.6 – 2.8)
Normal high-resolution impedance manometry	10 (42%)
Weak resting lower esophageal sphincter tone	5 (21%)
Ineffective esophageal motility	5 (21%)
Esophagogastric junction outflow obstruction	1 (4.2%)
**Ambulatory 24 hr pH study conducted**	17 (71%)
DeMeester Score [median (IQR)]	37.7 (4.1 – 104.5)

IRQ, interquartile range

Table 2. Impedance values at baseline and during spirometry study (n=22)

	Mean (kΩ) ±SD			
**Location**	**Baseline (kΩ)**	**Spirometry Set 1 (kΩ)**	**Recovery Period (kΩ)**	**Spirometry Set 2 (kΩ)**
Hypopharynx	3.59 ±2.20	2.35 ±1.68*	2.64 ±1.99*	2.28 ±1.75*
Pan-esophagus	2.52 ±0.74	1.57 ±0.59*	1.60 ±0.57*	1.37 ±0.67*^#

*Paired t-test against Baseline $p<0.001$; ^Paired t-test against Spirometry Set 1 $p<0.01$; #Paired t-test against Recovery Period $p<0.05$

Table 3. Analysis of spirometry set parameters (n=16).

	mean ± SD			
**Parameter**	**Spirometry Set 1**	**Spirometry Set 2**	**Difference**	***p* value***
FVC (L)	3.18 ±1.03	3.14 ±1.03	0.04 ±0.10	0.167
FEV_1(L)	2.45 ±0.93	2.31 ±0.79	0.14 ±0.24	0.042
PEFR (L/s)	5.21 ±1.65	4.54 ±1.42	0.67 ±1.15	0.034
EDI^	47.7 ±12.8	54.1 ±20.5	6.4 ±7.7	0.047

*Paire[illegible]est; ^EDI threshold >50 indicate anatomic laryngotracheal stenosis (sensitivity 95.9% and specificity 94.2%)

Figure 1 Click here to access/download;Figure;Figure 1.pdf

1. Pre-spirometry baseline

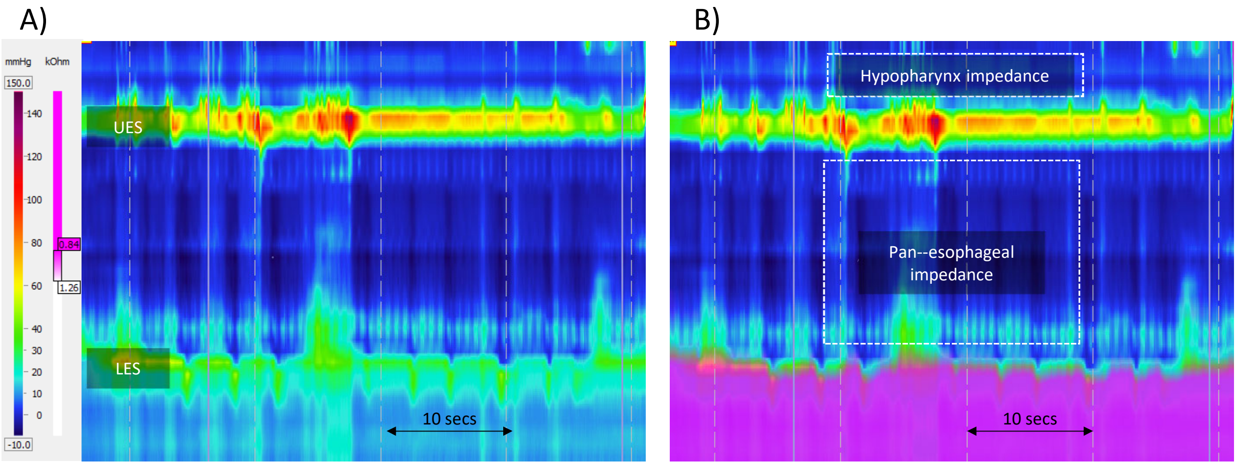

2. Forced vital capacity maneuver during spirometry set 1

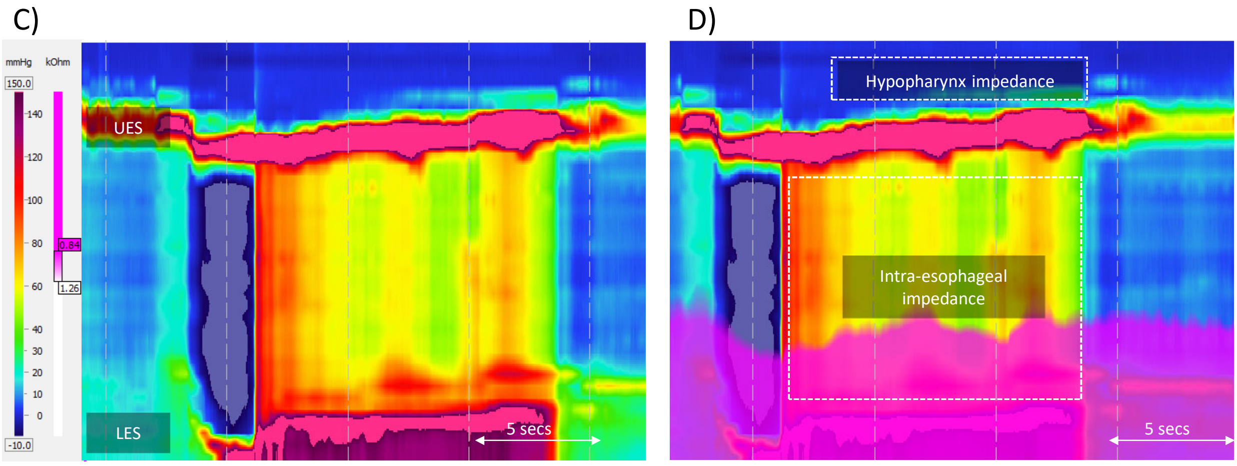

3. Post-spirometry recovery period

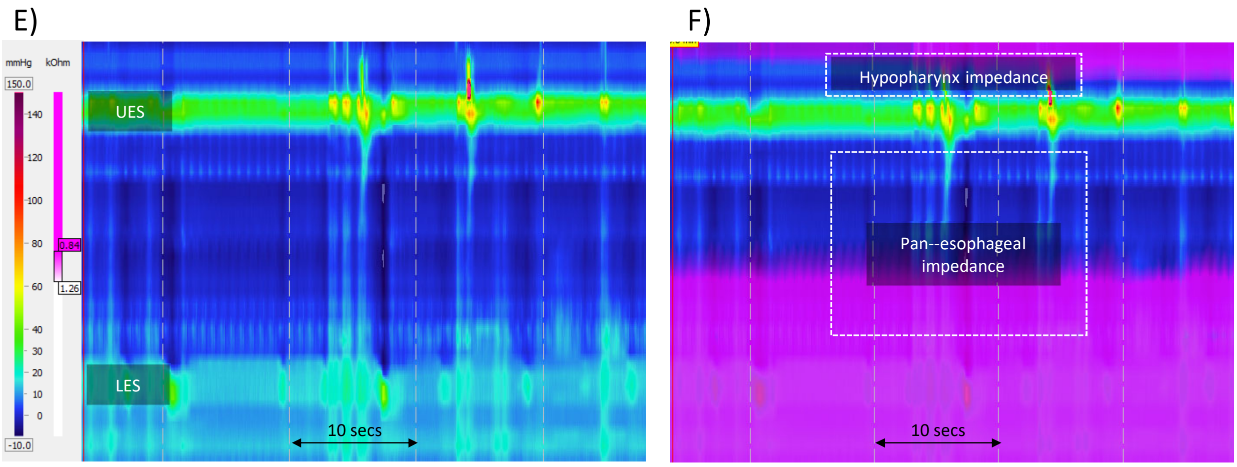

4. Forced vital capacity maneuver during spirometry set 2

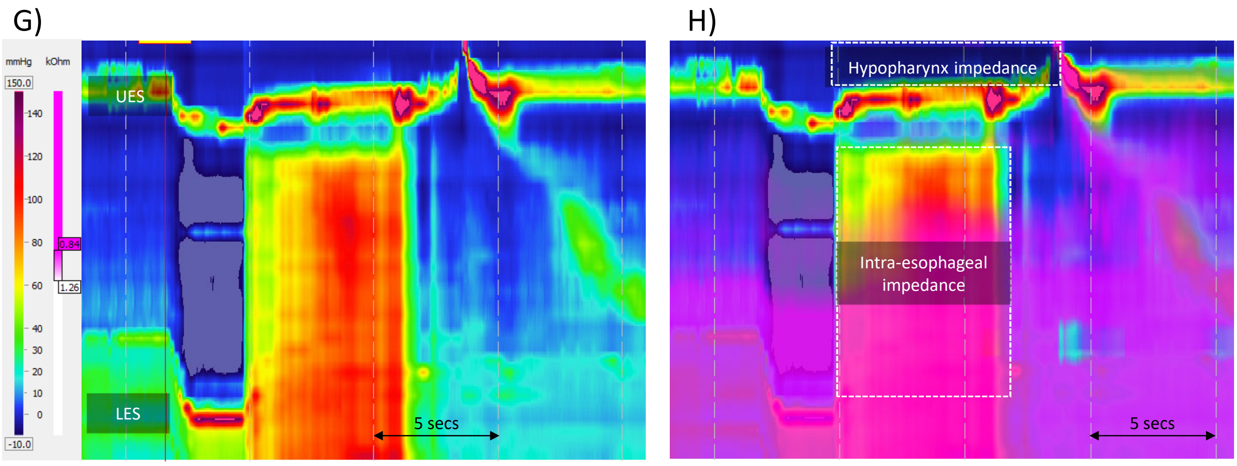

Figure 2

Click here to access/download;Figure;Figure 2.pdf

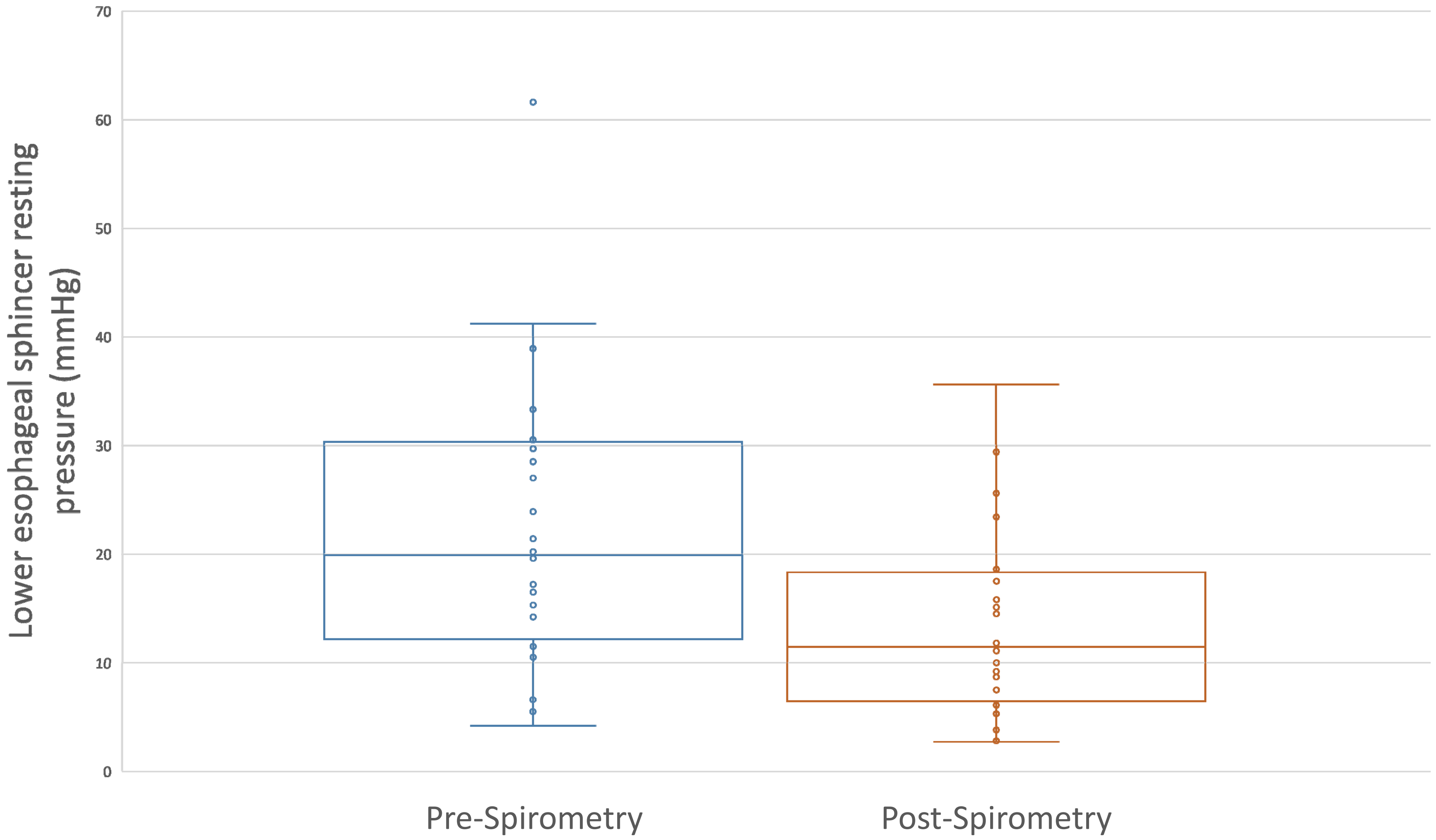

Supplement: Attachment — Submitted filename: PONE-D-23-07360.pdf [file pone.0291445.s002.pdf]
